# Ten simple rules for women principal investigators during a pandemic

**DOI:** 10.1371/journal.pcbi.1008370

**Published:** 2020-10-29

**Authors:** Pamela K. Kreeger, Amy Brock, Holly C. Gibbs, K. Jane Grande-Allen, Alice H. Huang, Kristyn S. Masters, Padmini Rangamani, Michaela R. Reagan, Shannon L. Servoss

**Affiliations:** 1 Department of Biomedical Engineering, University of Wisconsin-Madison, Madison, Wisconsin, United States of America; 2 Department of Biomedical Engineering, University of Texas at Austin, Austin, Texas, United States of America; 3 Microscopy and Imaging Center, Texas A&M University, College Station, Texas, United States of America; 4 Department of Bioengineering, Rice University, Houston, Texas, United States of America; 5 Department of Orthopaedics, Icahn School of Medicine at Mount Sinai, New York, New York, United States of America; 6 Department of Mechanical and Aerospace Engineering, University of California San Diego, San Diego, California, United States of America; 7 Center for Molecular Medicine, Maine Medical Center Research Institute, Scarborough, Maine, United States of America; 8 Ralph E. Martin Department of Chemical Engineering, University of Arkansas, Fayetteville, Arkansas, United States of America; Dassault Systemes BIOVIA, UNITED STATES

## Introduction

In the spring of 2020, nearly all academic institutions went to some level of shutdown/quarantine in order to slow the spread of Severe Acute Respiratory Syndrome Coronavirus 2 (SARS-CoV-2), the virus that causes Coronavirus Disease 2019 (COVID-19). For many universities, courses were moved online, laboratory-based research was required to slow or stop, and most on-site work shifted to telework. Optimistically, many academics thought initially that this might lead to a surge in research productivity. Indeed, by this point, we suspect that readers have heard that Isaac Newton apparently figured out calculus while in isolation during the plague. Consistent with this, some of the authors experienced or observed messaging from department chairs, center leaders, or mentors telling principal investigators (PIs) that the pandemic situation has likely created “extra time” for them to focus on writing grants and developing new ideas. Further, well-intentioned suggestions included ideas to shift research projects from experimental to computational questions; however, such shifts may represent a major research pivot for members of the lab group and require substantial support from the PI. Even as the pandemic persists and university leaders consider how to safely reopen labs and return to fall courses, which will result in new upheaval to our lives, there are still messages that quarantine might give scientists time to pursue new interests or work on long-forgotten projects [1].

However, if scientific fields are seeing increased output due to time focused on writing/computer-based tasks (e.g., publication submission, patent applications, and grant proposals), all indications suggest that this has been a benefit for men in science, and not women [2–4]. Discussions of these data have focused primarily on the fact that women do a disproportionate amount of house and childcare [5–7], and options used to provide support for this unpaid work have essentially evaporated (e.g., limiting outside workers into the home for cleaning, day cares not accessible to children of nonessential workers, and school and summer camp closures). Indeed, productivity gaps are being observed for working women in many industries and at a broader level have been suggested to be an issue for working parents regardless of gender due to the lack of school/day care options [8].

While we recognize that the impacts of COVID-19 are particularly acute for women with significant childcare/eldercare duties, we note that women PIs in academia carry disproportionately higher teaching and service loads [9–11]. These roles have been repeatedly emphasized by university leadership as essential in supporting students through the pandemic. For example, the shift to online teaching requires faculty to develop new teaching methods and provide additional support to students facing challenges with remote learning. Increases in service work have been noted by authors who serve in general advisory roles to undergraduate/graduate students (e.g., program directors). Additional work has been required from various research committees (e.g., biosafety and human subjects research) to support a safe return to research. While undoubtedly true that this work is essential, there has not been recognition that this requires more time from PIs and further disadvantages women from maintaining their research activity on par with male colleagues. Through a series of online discussions over the past few months, the authors have tried to identify “10 Simple Rules” to help women PIs navigate the pandemic. We recognize that these rules will not adequately address the additional challenges affecting women who also may suffer impacts due to other aspects of their identity (e.g., Black, Latina, and Indigenous women or LGBTQIA individuals).

Throughout this piece we will use several terms that may have slightly different meanings in different university structures. Our suggestions are aimed toward women PIs, meaning women who have an independent research position; these women may or may not lead a larger group or have a didactic teaching role. We use the term “students” to refer to undergraduate or graduate students that are being taught in a didactic setting and “trainee” to refer to undergraduate/graduate students or postdocs being mentored in a research setting. Research staff would include lab personnel such as technicians and scientists that are not independent PIs. Many PIs conduct research with both trainees and research staff; therefore, the term “group” implies all people that the PI mentors and manages. Finally, the term “staff” refers to administrative staff, whether in support of the research or teaching missions of the university.

### Rule 0: There are literally no rules

That’s right—the authors are going to acknowledge up front that there are no hard rules for a situation that has been described as “unprecedented” an unprecedented amount of times. Quite simply, the challenges the authors are facing may not reflect challenges others are facing because of differences in career stage, family situation, health stresses, degree that COVID-19 has impacted their locality, or many other variables. We instead are offering 10 suggestions that we hope will be useful or adaptable to others. In addition, we present suggestions to university leadership regarding institutional policies that can better support women PIs—now and in the future. Many of these suggestions are presented in more depth in [12]—we are indebted to the University of Wisconsin Caregiving Taskforce for authorizing us to amplify their efforts.

### Suggestion 1: Find a peer group of women to provide professional support

It is well established that peer networks are important for women in science, technology, engineering, and mathematics (STEM) [13]. The authors are all members of an online group for women PIs in biomedical engineering, which has historically served as a sounding board for professional concerns. However, many have their own smaller networks composed of women in their department/college/university, from similar racial/ethnic backgrounds, or with similar family situations (e.g., single parents, children with special needs, and eldercare responsibilities). We have found that maintaining these connections has been essential over the course of our careers and are even more important during this quarantine period. Examples to maintain connectivity included having a virtual happy hour/lunch, sending messages/e-mails to show support or to share your latest frustrations, or having a socially distant get-together. It is important to keep an eye out for women who seem to be struggling (e.g., not engaging in normal exchanges and sharing their stress) and reach out to them; likewise, if you are struggling, you should engage your network. Knowing you aren’t alone and that your concerns are valid is worth the time, as hard as it is to find the time.

#### Institutional suggestions

Create and/or support professional groups for women PIs. Examples include Association for Women in Science (AWIS), Society of Women Engineers (SWE), or individualized programs such as the Women Faculty Mentoring Program at the University of Wisconsin-Madison [14]. Provide financial support or relief from service to enable women to participate in such groups. Wherever possible, these groups should provide networks based on both professional interests and personal/identity concerns.

### Suggestion 2: Say no to requests to do anything outside of your main responsibilities

Despite the suggestion that you have gained time from things like losing your commute, recognize that you have lost far more time from challenges teaching online, working with your research team remotely, loss of childcare, and simple annoyances like finding toilet paper. So cut yourself some slack, and realize you won’t be able to do as much as you normally do. Perhaps you say no to peer review requests. Maybe you pass on a request to serve on a new committee. As things normalize, you can return to these tasks if they are important to you. Alternatively, if you can’t do them now, you can offer a time when you might be able to do them; the requesting party will need to recognize that your future schedule could go through upheaval as surges of the disease hit different areas and restrictions change. See S1 File for example responses.

#### Institutional suggestions

Cancel all nonessential service such as assessment reports that can be resumed after the pandemic with minimal effect. Suspend the tenure clock for probationary faculty. Support women PIs who need to cancel external service work (e.g., editing and reviewing) by making clear this will not be weighed in future promotion decisions. Conduct assessments of teaching and service loads within departments to achieve equity.

### Suggestion 3: Drop something

Recognizing that you have had to take on extra work (e.g., mentoring graduate students dealing with isolation and teaching third grade to your child), something has to give. In the best case, you would look at your workload and see something that really isn’t that important, takes up a lot of time, and you don’t enjoy. If so, drop this task! Alternatively, perhaps there are tasks that are necessary but could be delegated to a group member or staff who needs telework assignments. Provided your expectations are reasonable and you are able to provide mentoring support, accelerating others’ competency in tasks you normally reserve for yourself (e.g., proposal writing and budgeting) has the potential benefits of giving group members and staff a greater sense of ownership of the research mission, while reserving your time for critical tasks. Similarly, if your class has a teaching assistant (TA), engage them to help you improve the transition to virtual learning—insight from their own experiences taking such classes could be very beneficial. More often, this will mean not completing something you wanted to do but is not mission essential—unfortunately for many women, this has meant not submitting a half-finished proposal or pushing off writing a paper. So, we encourage you to look at your teaching/service load to see if there are items that can be put off or not done; you may find the questions posed in [15] useful as you determine what to keep or take on as your career progresses.

Or, consider adjusting your expectations for what your finished manuscript looks like—as we know, “perfect is the enemy of submitted.” Likewise, look at your homelife and do the same. True, your house might not be as clean as you would like, and you may be eating more frozen pizza than you normally would tolerate. Remember that you are not the only person accepting this as your “new normal.” We can’t forget that the goal during a pandemic is survival—if you are keeping yourself physically and mentally healthy, you are more than succeeding. Acknowledging these changes consciously and trying to make choices about where you make cuts might provide a semblance of control over a situation that has felt out of control.

#### Institutional suggestions

Reevaluate tenure/promotion expectations, extend the tenure clock, and formalize changes in writing. Provide adequate TA/grader support for classes. Make course evaluations, which are often susceptible to bias, optional. Alternatively, if course evaluations are done, make them developmental rather than evaluative in nature. Suspend requirements for probationary faculty to undergo peer teaching evaluations.

### Suggestion 4: When you have energy to do more than the minimum, use that in support of women and underrepresented groups

This may seem in conflict with prior suggestions—and it is—but women know they are able to pursue their scientific careers due to the hard-fought battles by the women before them [16]. Losing ground due to COVID-19 is a real possibility. We recognize that advocacy of this nature is a privilege, and not everyone is able to do so safely. If you are in the position to support women and underrepresented groups and have the energy, pick a cause and lean into it. Also, recognize that this action can take many forms, some of which may be a better fit for your individual situation. As examples of larger/more public actions, you could lobby your institution for policies to address the pandemic gender-related gap due to caregiving burdens [17], or push funding agencies to close racial disparities [18].

Remember that small actions add up—perhaps you don’t have the mental focus right now for a big battle or a position on a new committee. But if you are able to send an e-mail to colleagues and administrators pushing for more equitable policies from the university, you are contributing. You can share the names of scientists who are women, immigrants, and members of underrepresented groups with your trainees to make sure their work gets recognized and cited (for example, see the soon to be launched http://citeblackauthors.com). Take the time to incorporate Black and other underrepresented scientists in your teaching and research group meetings so the next generation recognizes their accomplishments. And for white men and women reading this piece, remember that service related to diversity and equity too often falls on the small number of PIs of color [19].

#### Institutional suggestions

Ensure that faculty and staff who contribute to diversity, equity, and inclusion initiatives are compensated for their time and effort in these areas, and this work is included in considerations of overall workload. Value these efforts in tenure and promotion decisions. Invite women and underrepresented PIs to present their work and provide an honorarium to cover caregiving costs for the time they will need to prepare and present virtually so that they can fully engage.

### Suggestion 5: Remember, you know yourself best

Maybe you thought you would have time to pick up a new hobby (or finish the many unfinished projects you already have). Six months (or more) into COVID-19, you have likely accepted that this is unrealistic. However, you know the things that have historically helped you relieve stress. Make a list of 10 of them. Some of them may not be an option during quarantine (oh, how some of us miss writing in coffee shops). But for those that are an option, try to do 1 of them every now and then. Maybe you like baking—bake cookies and drop them off on your colleague’s doorstep as the baking bandit (or just eat them all, no judging). Perhaps a daily walk is your relief, or you can join a yoga class online. You may personally find that taking a break from social media is calming. It doesn’t have to be every day, it doesn’t have to be long periods of time—but you have to find time for your mental health.

In the same manner, you know your group’s research strengths best. It is understandable during a global pandemic to feel called to shift your research priorities to the urgent problems at hand. Depending on your research skills, this may be a logical area for you to pursue and well worth the investment. For example, 1 of the coauthors was doing a sabbatical during the outbreak at a small biotech and participated in the development of a new diagnostic test. While not her original plan, the skills and collaborations she gained during this shift have opened new areas for her academic work. However, if your expertise is not relevant to infectious disease, diagnostics, personal protective equipment (PPE) testing, or other COVID-19 topics, it is perfectly appropriate to maintain your research focus. After all, there are many important problems that will remain even once the pandemic ends—and in some cases, they may have reached new levels of importance based on lack of preventative care during the pandemic or complications found in COVID-19 survivors.

#### Institutional suggestions

Offer employees access to programming that supports wellness, including aspects of both physical and mental health. Maintain a balance of support for research related to COVID-19 as well as previously established priorities.

### Suggestion 6: It’s OK to push back

Academia often seems to demand that you should be working at all times. And for those in academia who do not have significant demands at home due to childcare or eldercare, it is possible that the pandemic might be a time of productivity due to relief from some of the daily interruptions of working on-site. However, perpetuating the myth that we can all work to the same degree (or better!) than we did a few months ago is very damaging to many women PIs. When you hear statements such as “everyone is writing more grants now” and “since we have more time, let’s have a virtual conference about this topic,” it’s more than OK to push back that this is not your reality, regardless of the reason. You are likely to hear your voice amplified by others who were nervous to speak up. It is time for us to instead ask the person stating this to take on some of the work you have had to pass on (Suggestions 2 and 3), as a more equitable working environment should be a goal for all in science (Suggestion 4). If they balk, this is a perfect time to engage your network to vent your frustration (Suggestion 1). We have provided some sample responses we have used (S1 File).

#### Institutional suggestions

Provide training for department chairs and supervisors underscoring the strains that women and primary caregivers will face during the fall and spring semester, paying particular attention to how this crisis will be amplified for single parents, people of color, and others at the intersections of marginalized identities. Facilitate an education campaign for colleagues and students highlighting the immense strains on women and caregivers this fall, to improve empathy for delays in responses or slower completion of tasks. Normalize that children may appear during lectures or meetings. Be flexible about meeting times and attendance for committee service since faculty may be juggling multiple responsibilities at home.

### Suggestion 7: Remember, you have some flexibility to make your own schedule

If there are pockets of time where you find yourself able to focus better than others, do your best to protect them. Block these times on your calendar—both in the near future and in the upcoming months by declining invitations for extraneous responsibilities (Suggestions 2 and 3). Keep a “to-do” list of small tasks handy for those times that you have just a few minutes or you have time but not the mental energy to tackle a major project. You may find that knowing you will “get to it” is enough to clear some mental bandwidth to deal with your stress.

Conversely, if there are times your family needs your attention, put it on your calendar to prevent meetings from being scheduled in your family time. You may have colleagues that approach their telework or family scheduling very differently from you; do not put pressure on yourself if your approach radically deviates from those of your peers. Again, trust yourself to make the schedule and decisions that work best for you (see also Suggestion 5). Respect this right in your trainees as well—we can change the scientific culture if we reflect our values. Allow them to set their own schedules to fall outside the traditional workday window if that is a better fit for their situation (indeed, with social distancing in many labs, this may even be a necessity). And yes, it is okay to decline meetings that are requested at short notice (S1 File).

#### Institutional suggestions

Offer remote work and online teaching options for all faculty, instructors, and staff. Provide explicit policies that detail how faculty and instructors who teach face to face can pivot to asynchronous teaching modes and flexible work-from-home policies if an emergency arises. Utilize polling methods to identify meeting times rather than relying on meeting times from prior semesters when childcare was available during standard business hours—and remember that meal times and bed times may be particularly challenging for caregivers.

### Suggestion 8: Whatever help you can get, take it

This might seem obvious, but sometimes when we are overwhelmed, it’s hard to see the options that are there. Help with work tasks may come in the form of engaging staff who have limited telework—providing them with work may help them to avoid furlough, teach them new skills, and potentially lead to lasting support. For those with caregiving, help might come in the form of a family member who can provide childcare or screen time–based rewards that give you focused time to work. With many partners also working from home, discussions about the distribution of domestic and childcare responsibilities may be warranted to ensure equity and the ability of both partners to pursue their careers. Perhaps your kids are old enough that they can even help with some of your work—1 of the authors tried (unsuccessfully) to engage her son in doing analysis on ImageJ (NIH, Bethesda, Maryland). Another purchased a 3D printer and recruited her daughter to help print parts for an OpenSPIM setup. Maybe this is the time to get your children more involved in housework and cooking. Of course, these kinds of changes may be an uphill fight so take them on in slow steps when you are ready to deal with the next battle. Until then, refer to Suggestion 3 as often as needed.

#### Institutional suggestions

Create solutions beyond Family and Medical Leave Act (FMLA) for emergency leave and workload reductions. Offer a combination of creative solutions such as a 1-semester teaching release or course reduction, a 50% work option, etc. Repurpose travel funds to subsidize emergency childcare or eldercare. Offer a sick-day bank that allows others to contribute excess sick days to those in need. Utilize human resources (HR) and School of Education to provide a database of local resources for caregivers and resources for families homeschooling, tutoring, and support for families with children who need additional accommodations.

### Suggestion 9: Do your best to remember that others are struggling too—be empathetic and work to build a community

In times of stress, human beings are wired to focus on themselves—the fight-or-flight instinct is not about saving the community after all. This built-in response, coupled with the natural isolation of a quarantine, is a serious challenge to overcome. Finding virtual or socially distanced ways to maintain a sense of community with your friends and family are essential at this time. As a PI, you may want to arrange such events for your research group; many of the authors have done so and have found these to be appreciated by our groups even though the activities were not elaborate or lengthy. However, as you interact with others, it can be easy to fall into the pattern of comparing your stresses and deciding that you either have it “worse” or feeling guilty because you have it “better.” For example, while the authors have stresses related to the pandemic and gender equity imbalances, we acknowledge that our Black colleagues are dealing with racism and microaggressions on a daily basis. Women with different family situations (e.g., single parenting) will be dealing with different challenges. All of these feelings of stress are real and valid. Given the complex nuances of each individual’s situation, we will all benefit from trying to be more considerate as we work together.

So how do you remain considerate without falling into the trap of taking on the tasks that your lab group or staff should be responsible for? This is indeed a challenge that many woman PIs are dealing with—additional directives from university administration combined with lower productivity from their group and staff are putting the PI into a squeeze. We suggest that each situation is approached with empathy, while maintaining your standards and accountability. For example, empathy may mean that when you assign a task to a group member or staff, you ask them whether the timeline is feasible. If it’s not, that may be a sign that in the future you should aim to give them more advance notice. If a pattern of not completing work continues, it is then time to ask for an explanation. Recognize that just as no one is fully aware of your situation, you are not completely aware of your colleague’s situation. Those with younger children may struggle with finding focused time to work, while colleagues with older children may be dealing with remote school and developmental challenges that are unique to socially distanced teenagers. Perhaps you can help the group member or staff using some of the suggestions above to help them carve out work time and focus on the most important items. This is also a point to examine your role in this dynamic—are your expectations reasonable, or should they be modified? This may be an area where your network from Suggestion 1 can provide you honest feedback.

However, if you find that the group member or staff is not able to work through their challenges, it may be time to ask them to consider the impact of this situation on their mental health. Depression and anxiety often manifest in the inability to initiate or complete work tasks at the level an individual can typically perform at. Some group members and staff may share their struggles with you; indeed, studies have shown that students expect women faculty to be more accommodating of student challenges than men [20]. We know from our experiences that this takes up significant time/mental energy, and we suggest that you direct students, group members, and staff to university and community resources. If the situation persists, the next step may be to work with your HR to arrange for a leave (ideally, one that maintains benefits for the person).

#### Institutional suggestions

Provide leave options that maintain benefits for people at all levels at the university. Support and expand mental health care options, and regularly advertise these resources. Create funds to subsidize childcare costs for trainees.

### Suggestion 10: Don’t lose your sense of humor

We know, there is nothing funny about this situation. Many of us have needed or will need space to grieve deeply. However, our experience is that where you can share a laugh, you should. In that spirit, we offer a few of the more “tongue in cheek” suggestions that our larger peer group shared during discussion of this paper (S2 File).

#### Institutional suggestions

Participate in social media in ways that build community and provide moments of occasional levity.

## Concluding thoughts

We acknowledge that as academics we are privileged in this time of economic uncertainty, as most universities have been able to find ways to continue to pay salaries for PIs. Additionally, we are grateful for the rapid response from many universities that immediately offered tenure clock extensions as an acknowledgment that this crisis will impact junior faculty’s research progress the most heavily (although we would note that a simple solution of providing each junior faculty the same option may not provide equity). However, despite these policies, every author of this paper has hit her individual breaking point at least once during this crisis. So, while we don’t have all the answers ourselves, we hope that our thoughts will empower those reading this to realize that they are not alone and that saying no is an option available to them to minimize burnout. We also hope that this article, along with other commentaries [21], will provide fodder for departmental, college, and university leadership to consider how best to support scientists as they navigate these uncertain times.

While gender disparities are not a new topic in STEM, times of acute stress such as the COVID-19 pandemic have the ability to magnify the impact of these issues. Normalizing conversations around work–life balance, including equitable distribution of teaching/service duties and challenges with caregiving roles, is an essential step for the scientific community to realize a vision of equity. As we face the challenge of helping our students, group members, and staff through this pandemic, we challenge universities to assign additional value to teaching and mentoring, work that has been disproportionately shouldered by women in the academy [22]. In the best case, we will take what we learn from this unprecedented challenge to generate a more equitable and welcoming environment for women PIs.

## Supporting information

S1 File Example responses for declining requests or asking for accommodations. (DOCX)

S2 File Additional humorous “rules” for women principal investigators during this challenging time. (DOCX)

## References

[1] Goh HH, Bourne PE. Ten simple rules for researchers while in isolation from a pandemic. PLoS Comput Biol. 2020;16(6):e1007946 Epub 2020 Jun 26. 10.1371/journal.pcbi.1007946 32584810 PMC7316224

[2] Andersen JP, Nielsen MW, Simone NL, Lewiss RE, Jagsi R. COVID-19 medical papers have fewer women first authors than expected. Elife. 2020;9 Epub 2020 Jun 17. 10.7554/eLife.58807 32538780 PMC7304994

[3] Flaherty C. No room of one’s own: early journal submission data suggest COVID-19 is tanking women’s research productivity. Inside Higher Ed. 2020 4 21.

[4] Kitchener C. Women academics seem to be submitting fewer papers during coronavirus ‘Never seen anything like it,’ says one editor. The Lily 2020 4 24.

[5] Minello A. The pandemic and the female academic. Nature. 2020. 10.1038/d41586-020-01135-9 32303729

[6] Chou S, Desai T, Dixon E. Bay Area must enable economic, equitable recovery. San Francisco Chronicle. 2020 May 26.

[7] Miller CC. Nearly half of men say they do most of the home schooling. 3 percent of women agree. The New York Times. 2020 May 6.

[8] Perelman D. In the Covid-19 economy, you can have a kid or a job. you can’t have both. New York Times. 2020 Jul 2.

[9] Gibney E. Teaching load could put female scientists at career disadvantage. Nature. 2017.

[10] Guarino CM, Borden VMH. Faculty service loads and gender: are women taking care of the academic family? Res High Educ. 2017;58:672–694.

[11] Misra J, Hickes Lindquist J, Holmes E, Agiomavritis S. The Ivory Ceiling of Service Work. American Association of University Professors; 2011.

[12] University of Wisconsin. Systemwide Task Force on Caregiving and COVID-19 in the University of Wisconsin System. Co-Chairs: Stephanie Rytilahti and Jennifer Schuttlefield Christus. 2020 [cited 20 Aug 2020]. Available from: https://consortium.gws.wisc.edu/caregiving-task-force/?fbclid=IwAR3Lb3nMdvMXimhdrejLA8WTmtvDEq6LLkDjzEzVb5TEzDMub15WamjJm9w.

[13] Nokkala T, Culum B, Furnasoli T. Early career women in academia: an exploration of networking perceptions In: Eggins H, editor. The Changing Role of Women in Higher Education. Springer; 2016.

[14] Crone WC. Survive and Thrive: A Guide for Untenured Faculty. Morgan and Claypool Publishers; 2010.

[15] Carson T, Aguilera A, Brown S, Pena J, Butler A, Dulin A, et al A seat at the table: strategic engagement in service activities for early-career faculty from underrepresented groups in the academy. Acad Med. 2018;94(8):1089–1093. 10.1097/ACM.0000000000002603 PMC6626695 30649021

[16] Rossiter M. Women Scientists in America: Struggles and Strategies to 1940. Johns Hopkins University Press; 1982.

[17] Rytilahti S, Schuttlefield CJ. WGSC Statement Regarding Caregiving Recommendations For Fall 2020 Return To Campus. 2020 [cited 2020 Jul 8]. Available from: https://consortium.gws.wisc.edu/wp-content/uploads/sites/368/2020/06/WGSC-Caregiving-Recommendations-June-23-2020.pdf.

[18] Lorsch J, Gibbs K, Gammie A. What Can We Do to Combat Anti-Black Racism in the Biomedical Research Enterprise? 2020 Available from: https://loop.nigms.nih.gov/2020/06/what-can-we-do-to-combat-anti-black-racism-in-the-biomedical-research-enterprise/?fbclid=IwAR22yF3eoguOaMqqMfGHmxPwOdPfNqlj8rZlW_YnJOCUxqgmkKrBGaTaneU.

[19] Harley D. Maids of academe: African American women faculty at predominately white institutions. J Afr Am Stud. 2008;12(1):19–36.

[20] El-Alayli A, Hansen-Brown A, Ceynar M. Dancing backwards in high heels: female professors experience more work demands and special favor requests, particularly from academically entitled students. Sex Roles. 2018;79:136–150.

[21] Malisch JL, Harris BN, Sherrer SM, Lewis KA, Shepherd SL, McCarthy PC, et al Opinion: in the wake of COVID-19, academia needs new solutions to ensure gender equity. Proc Natl Acad Sci U S A. 2020;117(27):15378–15381. Epub 2020 Jun 20. 10.1073/pnas.2010636117 . 32554503 PMC7354923

[22] Park S. Research, teaching, and service: why shouldn’t women’s work count? J High Educ. 1996;67(1):46–84.

