# Supplementary material for: Ten simple rules for women principal investigators during a pandemic
Source: PLoS Comput Biol. 2020 Oct 29;16(10):e1008370. doi: 10.1371/journal.pcbi.1008370 (PMC7595267; doi:10.1371/journal.pcbi.1008370)
Supplement: S1 File — (DOCX) [file pcbi.1008370.s001.docx]

**Supplemental File 1**

Responses that some of the authors have used:

*A general ‘no’ (you don’t have to provide reasons for everyone!)*

I am unable to take this on. Thank you for considering me.

*A general ‘no’ if you are open to doing in the future*

I am unable to take this on at this time. Thank you for considering me. It is possible I may be able to do so in XX.

*In response to review requests*

Due to lack of childcare during COVID-19, I am taking on limited review requests.

or

Due to extra service duties during COVID-19, I am taking on limited review requests.

*In response to student committee requests that are short notice*

Most students whose committees I serve on and my own students reach out for scheduling well in advance. My calendar tends to book up very quickly and I rarely have an opening at less than two weeks' notice unless it is an emergency. It is not clear to me this is an emergency. In addition, summer is a difficult time to schedule faculty due to our focus on research efforts.  If you want meaningful input from your committee, you will need to plan ahead and give us enough options to select from and more time to respond.

*In response to the suggestion that a full-day virtual meeting should be scheduled*

The lack of childcare and/or extra clinical demands during COVID-19 would make this impossible for me to participate in. While I appreciate the idea, I think it would be better to wait for a time when we can have more equitable participation.

*In response to a proposal to hold a week-long virtual conference*

The idea of a virtual meeting is great -- but only if the attendees can fully engage. One of the things I love about meetings is to be able to focus on the science without other commitments. However, many scientists are unable to go into their offices either due to campus restrictions or the loss of childcare. So, while I completely understand the issues that you raise, such a meeting would knowingly limit participation from many of our attendees, and disproportionately impact younger (and in particular women) scientists with family obligations.

*In response to a request with an unreasonable timeline from a funding agency*

I have greatly valued my research support from XXX to support my work and the training of my graduate students and postdoctoral fellows. However, a challenge with receiving this support has been the reporting and administrative burden on PIs, often not commensurate with the effort originally stated in the grant. At best this is a small nuisance, but at worst it results in distraction from the research and resulting productivity in support of the mission of XXX.

A specific example is a recent request to report to the PO by phone within 24-48 hours on how projects will be managed during the COVID19 crisis. While I understand the need for a funding agency to know what we are able to do, the timeline is simply too short given that the PI must first examine the new regulations from the university to see what is possible, then communicate with each team member to confirm that they have the ability to continue the work that is allowed, and finally summarize this data in an interpretable manner for the PO.

In addition to allowing a longer timeline, I would also suggest that such requests are handled over email to give PIs (and program staff!) the flexibility to respond as their schedule permits. I am sure you are aware that many PIs are facing a sudden loss of childcare and having to move towards a more flex schedule of working - in the shared goal of maintaining a diverse workforce, I would ask the XXX to consider the challenges faced by PIs in their future administrative requests during the COVID19 crisis.
