# Supplementary material for: Ten simple rules for women principal investigators during a pandemic
Source: PLoS Comput Biol. 2020 Oct 29;16(10):e1008370. doi: 10.1371/journal.pcbi.1008370 (PMC7595267; doi:10.1371/journal.pcbi.1008370)
Supplement: S2 File — (DOCX) [file pcbi.1008370.s002.docx]

**Supplemental File 2**

Semi-serious responses from a larger group of BME women faculty. All provided permission to include their de-identified “tip”.

Rule X. Don't accept any invitations to do anything outside of your main responsibilities. (Damn...I just broke that one.) *this is one of the co-authors*

Rule X: Keep a pillow nearby to scream into every time a colleague sends you an email saying how great it is that we all have so much 'grant writing time' on our hands.

Rule X. Wine. The end.

Rule X: Video games no longer count toward screen time - but only IF you don’t argue with your sibling.

Rule X: Labor laws no longer apply and older children can now be research assistants and/or subjects.

Rule X: Train your kids to fake minor emergencies to get out of all.the.zoom.meetings.

Rule X: Service expectations are now completely fulfilled by raising scientifically literate humans of our own and science communication by way of family conversations, Facebook and Twitter debates.

Rule X. Make those who are shaming others to stop whining about their kids and “enjoy being with them” to give us 40 hrs of free babysitting.

Rule X: Don’t think of it as avoiding your children so you can work. You are teaching ‘independence’ and ‘unstructured play’.

Rule X: When your childcare facility unexpectedly closes, find a few compatible families to create a co-op with. Facilitate unsupervised transportation by digging underground tunnels between the families’ homes.

Rule X: Install a pull-up bar in your home. For each unjust headline or article you read, or frustrating email you receive, attempt/do pullup. Think of how buff you will be at the end of the pandemic.

Rule X: If you have a partner, insist they take on more care-taking responsibilities. If they decline, hold the wine hostage.

Rule X: Embrace Fight Club Mode: develop the art of letting that which can, slide. Consider allowing kids to play Fight Club to gain more time to work on the things that can’t slide.

Rule X: Order take out frequently, to “support local business”.

Rule X: Buy a large tea kettle/samovar to facilitate effortless on-demand supply of soothing or mildly caffeinated teas. For deadline emergencies, use instant coffee.

Rule X: Coffee. Chocolate. Wine. Repeat.

Rule X: There is no such thing as too many craft supplies if you have kids. In emergencies, provide them with delivery packaging so that it can be ‘upcycled’.

Rule X: Neighbors not understanding that you are socially distancing? Develop a powerpoint presentation to explain pandemics. Count as a broader impact on your next progress report.

Rule X: Look on the bright side. At least with masks on, we won’t get told to smile more in our teaching evaluations!

Rule X. Play COVID BINGO.

| Go to more than two stores to find toilet paper | Hear or use the term ‘unprecedented’ | Receive e-mail from a colleague’s concerned student | Pull out your sewing machine to become mask making fiend | Use more than one video platform in a day |
| --- | --- | --- | --- | --- |
| Child/partner/  pet interrupts  Zoom meeting (on your end) | Fill out teleworking timesheet | Forget you are unmuted and reply that ‘yes, you are still on the boring meeting’ | Told to “treasure being able to spend so much time with family/kids” | Do a call from your closet as it is the only place to escape in your house |
| Receive e-mail from concerned student | Colleague asks why you always seem so tired | Free Space | Told that teleworking timesheet is not needed | Asked to review a paper by a man while never finishing your own |
| Class reassigned to 7pm on Friday nights | Internet outage | Asked to be on another committee | Use ‘hybrid’ outside of genetics context | Refer to your black sweats as ‘dress pants’ |
| Use 10 minute break to fight the patriarchy and/or systemic racism | Purchase either wine or chocolate to cheer up a colleague | Child/partner/  pet interrupts  Zoom meeting (on other attendee’s end) | Colleague sends email about all the extra writing they are doing | Steal your chair/ monitor/standing  desk from your work office |
